# Treatment outcomes of meningitis and its associated factors among neonates admitted to public hospitals in Harar Town, Ethiopia: Cross-sectional study

**DOI:** 10.1371/journal.pone.0355843

**Published:** 2026-08-11

**Authors:** Getahun Tamiru Yirsaw, Tadesse Bekele Tafesse, Abera Jambo Bune, Shambel Nigussie Amare

**Affiliations:** 1 Clinical Pharmacy Unit, Hiwot Fana Comprehensive Specialized Hospital, Haramaya University, Harar, Ethiopia; 2 Department of Pharmacology and Toxicology, Haramaya University, Harar, Ethiopia; 3 Department of Clinical Pharmacy, School of Pharmacy, College of Health and Medical Science, Haramaya University, Harar, Ethiopia; 4 Discipline of Pharmacy, Faculty of Health, University of Canberra, Canberra, Australia; Maitama District Hospital, NIGERIA

## Abstract

**Background:**

Meningitis in neonates remains a major public health concern, particularly in low- and middle-income countries. Despite its high morbidity and mortality, evidence on treatment outcomes and determinants of poor prognosis is limited. This study aimed to assess treatment outcomes of meningitis and its associated factors among neonates admitted to public hospitals in Harar town, Ethiopia.

**Methods:**

A facility-based cross-sectional study was conducted among 506 neonates diagnosed with meningitis admitted between October 2020 and October 2024. Data were extracted from medical records. Treatment outcomes were categorized as good or poor. Binary and multivariable logistic regression analyses were performed to identify factors associated with poor treatment outcomes. Statistical significance was declared at a p-value <0.05 using adjusted odds ratios (AORs) with 95% confidence intervals (CIs).

**Results:**

A total of 169 (33%) neonates experienced poor treatment outcomes. Assisted vaginal delivery (AOR = 3.70; 95% CI: 1.42–9.69), positive cerebrospinal fluid (CSF) culture (AOR = 3.74; 95% CI: 1.76–7.96), elevated CSF white blood cell count >2500 cells/mm³ (AOR = 6.10; 95% CI: 2.54–14.69), high CSF protein >400 mg/dL (AOR = 5.43; 95% CI: 2.84–10.36), low CSF glucose <10 mg/dL (AOR = 3.57; 95% CI: 1.64–7.80), seizures at admission (AOR = 5.07; 95% CI: 2.71–9.46), seizures after admission (AOR = 3.88; 95% CI: 1.82–8.25), early-onset neonatal sepsis (AOR = 4.27; 95% CI: 2.03–8.99), and non-exclusive breastfeeding (AOR = 2.20; 95% CI: 1.06–4.57) were predictor of poor treatment outcomes.

**Conclusion:**

About one-third of neonates with meningitis experienced poor treatment outcomes. Factors associated with poor outcomes included assisted vaginal delivery, severe CSF abnormalities (positive culture, high white blood cell count, elevated protein, and low glucose), seizures at or after admission, early-onset neonatal sepsis, and non-exclusive breastfeeding. These findings highlight the need for early risk stratification and timely interventions to improve neonatal care and treatment outcomes.

## Introduction

Meningitis is a serious infection that causes inflammation of the subarachnoid space of the brain and spinal cord. It is usually caused by bacteria, viruses, fungi, or parasites [1]. Newborns are especially vulnerable due to immature immunity, with neonatal meningitis classified as early-onset (≤72 hours after birth) or late-onset (>72 hours) [2,3].

The pathogens causing neonatal meningitis depend on gestational age, postnatal age, and location [3]. Early-onset cases are mainly due to *Group B Streptococcus* (GBS) and *Escherichia coli* (*E. coli*) [2,4–7]. Late-onset pathogens vary by gestational age, birth weight, and healthcare setting: GBS and *E. coli* are common in community-acquired cases, while staphylococci, *Staphylococcus aureus* (*S. aureus*), and *Klebsiella* species are frequent in healthcare-associated infections [7–10].

Globally, the incidence and mortality rates of neonatal meningitis were estimated at 854 and 137.2 per 100,000 population, respectively, in 2019 [11]. The disease also imposes a substantial economic burden, with healthcare expenditures ranging from US$222 to US$33,635 per patient worldwide [12]. The incidence and mortality of neonatal meningitis are disproportionately higher in low- and middle-income countries, with 0.5–6 cases per 1,000 live births and mortality rates of 20–37%, compared to 0.3–0.4 cases per 1,000 live births and 2–10% mortality in high-income countries [5,6,13–18]. Among survivors, 20–50% develop long-term neurological sequelae, including hearing loss, blindness, epilepsy, hydrocephalus, subdural empyema, and psychomotor developmental delay [19–21].

Sub-Saharan Africa, part of the global “meningitis belt,” continues to bear a disproportionately high burden, with reported incidence and mortality rates including 96 per 1,000 live births in Kenya [22], and mortality rates of 24.0% in Angola [23], 29.4% in Tanzania [24], and 37% in Gambia [16]. Ethiopia ranks third globally in meningitis mortality, with treatment for bacterial meningitis costing a median of 98,812 ETB (≈US$3,593.2) per patient [25,26].

Multiple clinical factors have been identified as predictors of poor prognosis in neonatal meningitis, including seizures, elevated CSF protein, low birth weight or prematurity, early onset of disease, and low CSF glucose levels [18,19,27].

Predictors of neonatal meningitis outcomes can differ across populations and healthcare settings. Although the disease causes substantial morbidity and mortality in low-income countries, studies on treatment outcomes and prognostic factors are scarce, especially in Ethiopia. To the best of the author’s knowledge, only one Ethiopian study has examined this issue [28], but its small sample size and limited representativeness restrict the generalizability of its findings.

Given the scarcity of evidence and the high burden of disease, context-specific data are essential to improve neonatal care and clinical outcomes. Therefore, this study aimed to assess treatment outcomes of meningitis and its associated factors among neonates admitted to public hospitals in Harar town, Ethiopia. The main research question was: *Which clinical, demographic, and healthcare-related factors are associated with adverse outcomes in neonatal meningitis in this setting?* Given the limited prior data, this study is primarily **exploratory**, generating evidence to inform clinical practice and guide future confirmatory research.

## Materials & methods

### Study design and settings

A cross-sectional study design was conducted in Hiwot Fana Comprehensive Specialized University Hospital (HFCSUH) and Jugal General Hospital (JGH) from February 10 to March 20, 2025. Harar is the capital city of the Harari regional administration. It is located in the eastern part of the country, around 500 kilometers from the Ethiopian capital city, Addis Ababa. HFCSH was founded in 1948 and transitioned to a university-specialized hospital in 2010. JGH was founded in 1909. Both hospitals provide medical, surgical, pediatric, obstetric, and gynecologic services and have neonatal intensive care units.

### Study population and variables

All medical records of neonates admitted to the Neonatal Intensive Care Units (NICUs) of HFCSUH and JGH with a confirmed diagnosis of meningitis between October 1, 2020, and October 31, 2024, were included in this study. Records with incomplete information, such as missing diagnosis, treatment regimen, or discharge summary, as well as those who discontinued treatment within the first 72 hours, and those referred to other facilities, were excluded. Data were collected on socio-demographic factors (admission site, sex, age at admission, birth weight, and gestational age); clinical factors, including pre-existing medical conditions (congenital heart defects, Down syndrome, congenital hydrocephalus), maternal conditions and complications during delivery (history of premature rupture of membranes (PROM), meconium-stained amniotic fluid, and chorioamnionitis), and mode of delivery (spontaneous vaginal delivery, cesarean section, or assisted vaginal delivery (Forceps Delivery or Vacuum Extraction); diagnostic information such as symptoms at presentation, blood test results, CSF findings, type of pathogen, and specific isolated organisms; and treatment-related factors, including antimicrobial therapy (choice of antimicrobials) and supportive care (nutritional support and management of complications).

### Outcome measurement

In this study, treatment outcomes were categorized as either *good* or *poor*. Outcomes were assessed at the time of hospital discharge or in-hospital death, representing short-term in-hospital outcomes.

A good treatment outcome was defined as clinical recovery or improvement at discharge without documented complications. A poor treatment outcome was defined as in-hospital death, discharge without clinical improvement, or discharge with documented neurological complications as recorded in the treating clinician’s discharge summary.

### Sample size determination and sampling technique

A single-population proportion formula was used to estimate the sample size for the primary outcome variable, while double-population proportion formulas were applied to determine sample sizes for the associated factors. For the treatment outcome, the calculation was based on the assumptions of a 95% confidence interval, a 5% margin of error, and a previously reported proportion of poor outcomes (20.7%) from a study conducted at Tibebe Ghion Comprehensive Specialized Hospital in Bahir Dar, Ethiopia [28].


$$\begin{array}{c} {\text{n}}_{\text{i}}=\frac{\left(\text{Z}\alpha /\text{2}{)}^{\text{2}}\text{p}(\text{1}-\text{p}\right)}{{\text{d}}^{\text{2}}}\;{\text{n}}_{\text{i}}=\frac{\left(\text{1}.\text{96}{)}^{\text{2}}\text{0}.\text{207}(\text{1}-\text{0}.\text{207}\right)}{{\left(\text{0}.\text{05}\right)}^{\text{2}}}\;=\text{ 252} \end{array}$$

Sample size for associated factors (i.e., seizure [29], preterm [30], early-onset neonatal sepsis (EONS) [31], prolonged shock [32], mechanical ventilation [33], and congenital heart disease [34] were computed using Epi Info V.7. 1.4, with considered the following assumptions: a 95% confidence interval, a 5% margin of sampling error tolerated, and an 80% power.

The largest sample size obtained from the single- and double-population proportion calculations was selected as the final sample size. EONS yielded the highest estimate; therefore, it was used to determine the required sample size. After adding a 5% contingency to account for incomplete records, the final sample size was 506, and these 506 medical records were subsequently reviewed. Study participants from each hospital were selected using a simple random sampling method. First, the registration numbers of eligible neonates were obtained from the admission logbooks. These numbers were then entered into Microsoft Excel, and a lottery method was applied to randomly select the required records. Proportional allocation was used to distribute the sample across HFCSUH and JGH, based on the number of neonates admitted with meningitis during the study period (HFCSUH = 725 and JGH = 321). Accordingly, 351 records were selected from HFCSUH and 155 from JGH.

### Data collection tool and procedure

A structured tool based on relevant literature was used to extract socio-demographic, clinical, and treatment-related data from medical records. Eligible records from neonates admitted to the NICUs of HFCSUH and JGH between October 1, 2020, and October 31, 2024, were identified from registry logbooks, retrieved from the card rooms, and records meeting the eligibility criteria were retrieved and reviewed using the data abstraction tool. Data extraction was conducted from February 10 to March 20, 2025, ensuring completeness and consistency.

### Data quality assurance

Before data collection, a pretest was conducted with 5% of the sample at Haramaya General Hospital, and the data collection tool was modified based on the findings. Four clinical pharmacists were involved in the data collection process. Before data collection, they received appropriate orientation on the objectives of the study and the use of the data abstraction tool. Throughout the data collection period, the completeness and consistency of the extracted information were checked continuously and reviewed again before data processing to ensure data quality.

### Statistical analysis

Data were entered into EpiData version 7.2 to ensure accuracy and completeness, then exported to SPSS version 27 for analysis. Descriptive statistics were computed using frequencies and percentages for categorical variables and means with standard deviations for continuous variables. Binary logistic regression was first conducted to assess the association of each variable with treatment outcomes, and variables with p < 0.25 in the binary analysis were included in the multivariate logistic regression to identify independent predictors. The final model’s goodness of fit was confirmed using the Hosmer-Lemeshow test (p = 0.53), and multicollinearity was assessed using the variance inflation factor (VIF = 1.74), indicating no problematic correlations. Associations were reported as crude odds ratios (COR) and adjusted odds ratios (AOR) with 95% confidence intervals, and a p-value < 0.05 was considered statistically significant.

### Operational definitions

Neonatal meningitis was classified by diagnostic certainty into **confirmed, probable, and suspected** (**possible**) cases.

**Confirmed neonatal meningitis** was defined as the presence of clinical features suggestive of meningitis (e.g., fever, seizures, poor feeding, or thermal instability) together with microbiological evidence from CSF, including a positive CSF culture and/or a positive CSF Gram stain [35,36].

**Probable neonatal meningitis** were neonates presented with clinical features suggestive of meningitis and abnormal CSF findings, such as leukocyte count >32/mm³ in term and >29/mm³ in preterm neonates, glucose <34 mg/dL in term and <24 mg/dL in preterm neonates, or protein >170 mg/dL in term and >150 mg/dL in preterm neonates, but without microbiological confirmation [35,36].

**Suspected** (**possible**) **neonatal meningitis** is defined by the presence of clinical signs and symptoms consistent with meningitis, but CSF analysis was normal despite a high clinical suspicion [35,36].

**Neurological sequelae** were defined as any secondary conditions resulting from meningitis, including persistent seizure disorder after treatment, subdural effusion, brain abscess, hearing loss, hydrocephalus, and subdural empyema.

### Ethical considerations

This study was approved by the Institutional Health Research Ethics Review Committee (IHRERC) of the College of Health and Medical Sciences, Haramaya University, under reference number **IHRERC/041/2025,** for the study entitled *“Treatment Outcomes of Meningitis and Its Associated Factors among Neonates Admitted to Public Hospitals in Harar Town, Ethiopia: Cross-sectional Study.”* The study was conducted between February 10 and March 20, 2025. Official cooperation letters were sent to HFCSUH and JGH to facilitate access to medical records. Patients’ privacy and confidentiality were strictly maintained: no personal identifiers were recorded, and direct consent from parents or guardians was not obtained due to unregistered phone numbers and the absence of direct interaction or interviews. Institutional consent was obtained from the hospitals, and all data were handled confidentially to ensure that patients’ identities remained protected.

## Results

### Socio-demographic characteristics of patients

A total of 506 neonates with meningitis were included in this study, with 351 (69.4%) from HFCSUH and 155 (30.6%) from JGH. Of these, 278 (54.9%) were male, resulting in a male-to-female ratio of 1.22:1. The majority of neonates were in the early neonatal period (<7 days old), with a mean age of 8.91 ± 5.92 days. Regarding place of delivery, most neonates were born in specialized/tertiary hospitals (167, 33.0%) or health centers (152, 30.0%) (Table 1).

**Table 1:** Socio-demographic characteristics of neonates admitted with meningitis at public hospitals in Harar town, Eastern Ethiopia, from February 10 to March 20, 2025 [n = 506].

Variable	Category	Frequency	Percent
**Site of admission**	HFCSUH	351	69.4
	JGH	155	30.6
**Sex**	Female	228	45.1
	Male	278	54.9
**Age (days)**	<7	270	53.4
	7-28	236	46.6
**Place of delivery**	Home	9	1.8
	Health center	152	30.0
	Primary hospital	56	11.1
	General hospital	87	17.2
	Specialized/tertiary hospital	167	33.0
	Private health facility	35	6.9

HFCSUH: Hiwot Fana Comprehensive Specialized University Hospital; JGH: Jugal General Hospital.

### Neonatal and maternal characteristics of health

Among the neonates, 181 (36.2%) had coexisting sepsis at admission, including 116 (22.9%) with EONS; 65 (12.8%) with late-onset neonatal sepsis (LONS). The mean birth weight was 3099 ± 393 g, and the mean gestational age was 36.9 ± 1.13 weeks; 69 (13.6%) were low birth weight, and 130 (25.7%) were preterm. Pre-existing medical conditions were observed in 42 (8.3%) neonates. Most were delivered via spontaneous vaginal delivery (314; 62.1%). Maternal complications occurred in 119 (23.5%) cases, mainly PROM (71; 14.0%) and eclampsia (31; 6.1%) (Table 2).

**Table 2:** Neonatal and maternal health characteristics among neonates admitted with meningitis at public hospitals in Harar town, Eastern Ethiopia, from February 10 to March 20, 2025 [N = 506].

Variable	Category	Frequency	Percent
**Co-existing** **Illness**	EONS	116	22.9
	LONS	65	12.8
**Birth weight of neonate (grams)**	<2500	69	13.6
	≥2500	437	86.4
**Gestational age of neonate (weeks)**	<37	130	25.7
	≥37	376	74.3
**Pre-existing medical condition**	Yes	42	8.3
**Type of pre-existing medical condition**	Congenital hydrocephalus	35	6.9
	Down syndrome	4	0.8
	Congenital heart defects	3	0.6
**Mode of delivery**	C/S	153	30.2
	Spontaneous vaginal	314	62.1
	Assisted vaginal delivery (Forceps Delivery or Vacuum Extraction)	38	7.7
**Complications during delivery**	Yes	119	23.5
**Type of complication**	PROM	71	14.0
	Eclampsia	31	6.1
	Chorioamnionitis	11	2.2
	Meconium stain in amniotic fluid	6	1.2

C/S: cesarean section; EONS: early onset neonatal sepsis; LONS: late onset neonatal sepsis; PROM: premature rupture of membranes.

### Laboratory and CSF findings

The mean CSF white blood cell count was 1764.3 ± 833.3 cells/μL, with 70 (14.2%) neonates exceeding 2500 cells/mm³. Elevated CSF protein (>400 mg/dL) was observed in 118 (23.3%) neonates, while 79 (15.6%) had glucose levels below 10 mg/dL; mean CSF protein and glucose were 300.2 ± 108.2 mg/dL and 15.4 ± 6.9 mg/dL, respectively. Gram staining was positive in 113 (22.3%) cases, predominantly Gram-positive cocci (63; 12.5%) and Gram-negative rods (40; 7.9%). CSF cultures were positive in 71 (14.0%) neonates, with GBS in 30 (5.9%) and *E. coli* in 20 (4.0%). Hematologic abnormalities included thrombocytopenia in 35 (6.9%), anemia in 199 (39.3%), and leukocytosis in 134 (26.5%) neonates (Table 3).

**Table 3:** Results of laboratory findings and identified pathogens of meningitis among neonates admitted at public hospitals in Harar town, Ethiopia, from February 10 to March 20, 2025.

Variable	Category	Frequency	Percent
**CSF WBC cells/mm³**	<2500	422	85.8
	≥2500	70	14.2
**CSF Protein (mg/dl)**	<400	388	76.7
	≥400	118	23.3
**CSF Glucose (mg/dl)**	<10	79	15.6
	≥10	427	84.4
**CSF gram stain**	Yes	113	22.3
**Identified the pathogen from the Gram stain**	Gram-positive coccus	63	12.5
	Gram-negative rods	40	7.9
	Gram-positive rods	9	1.8
	Gram-negative coccus	1	0.2
**CSF culture**	Yes	71	14.0
**Isolated micro-organism**	GBS	30	5.9
	*E.coli*	20	4.0
	Klebsiella	16	3.2
	*S. aureus*	3	0.6
	*L. monocytogenes*	1	0.2
	Acinetobacter	1	0.2
**WCC (10** ^**9**^ **/L)**	<15	372	73.5
	≥15	134	26.5
**Hgb (g/dl)**	<11.5	199	39.3
	≥11.5	307	60.7
**Platelet count (10** ^**9**^ **/L)**	<150	35	6.9
	≥150	471	93.1

CSF: cerebrospinal fluid; EONS: Early Onset Neonatal Sepsis; GBS: Group B streptococcus; Hgb: Hemoglobin; LONS: Late Onset Neonatal Sepsis; WCC: White cell count.

### Clinical findings, treatment and outcome

**Fig 1:**
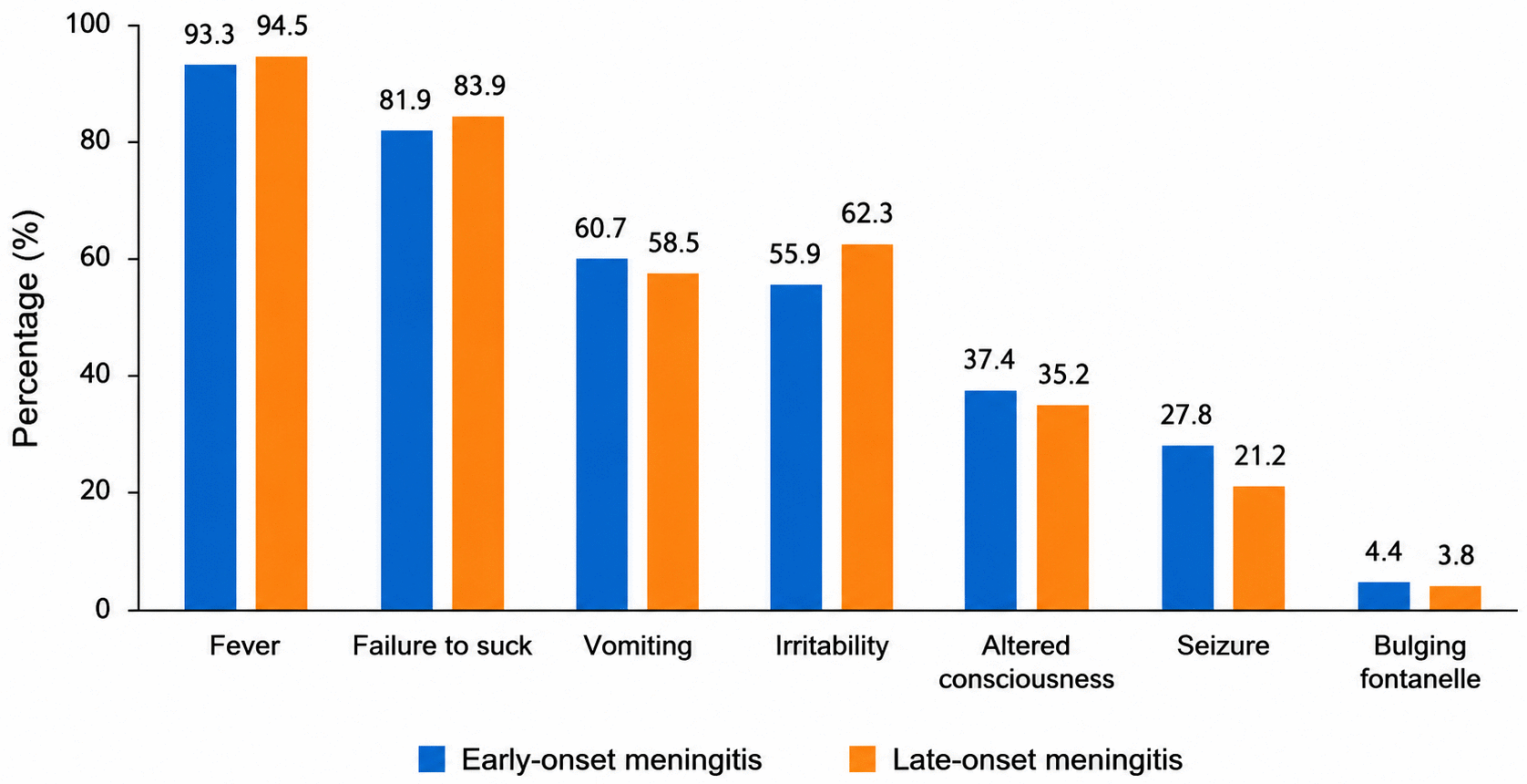
Signs and symptoms among neonates admitted with meningitis in public hospitals in Harar Town, Eastern Ethiopia.

The most common clinical manifestations among neonates with meningitis were fever (475/506, 93.9%), failure to suck (419/506, 82.8%), vomiting (302/506, 59.7%), and irritability (298/506, 58.9%) (Fig 1). Among the 506 neonates treated for meningitis, 416 (82.2%) received ampicillin plus gentamicin as the initial empirical antibiotic regimen, while 54 (10.7%) received ampicillin plus cefotaxime. During hospitalization, 143 (28.3%) neonates developed one or more complications, the most common being seizures (62/506, 12.3%), renal failure (17/506, 3.4%), and septic shock (16/506, 3.2%). Among the survivors, 73 (17.8%) were discharged with neurological sequelae, predominantly seizures (49/411, 12.0%) and hydrocephalus (11/411, 2.7%). Overall, 339 (67.0%) neonates had good treatment outcomes, whereas 167 (33.0%) had poor treatment outcomes, including 95 (18.8%) who died during hospitalization (Table 4).

**Table 4:** Complications of meningitis and treatment outcomes among neonates admitted at public hospitals in Harar town, Eastern Ethiopia, from February 10 to March 20, 2025.

Variables	Category	Frequency	Percent
**Complications developed after 72 hours of treatment**	Yes	130	25.7
**Complications developed in-hospital**	Yes	143	28.3
**Complications in the hospital**	Seizure	62	12.3
	Renal failure	17	3.4
	Septic shock	16	3.2
	Hydrocephalus	11	2.2
	Subdural effusion	11	2.2
	Respiratory failure	10	2.0
	Brain abscess	9	1.8
	Others*	7	1.4
**Discharged with complications**	Yes	73	17.8
**Complication at discharge**	Seizure	49	12.0
	Hydrocephalus	11	2.7
	Subdural effusion	5	1.2
	Weakness	5	1.2
	Others**	3	0.7
**Outcomes of treatment**	Poor	167	33.0
	Good	339	67.0

Others*: subdural empyema (3, 0.6%); weakness (2, 0.4%); brain infarction (1, 0.2%); ventriculomegaly (1, 0.2%). Others**: Hemiparesis (2, 0.47%); deafness (1, 0.23%).

### Factors associated with poor treatment outcomes

Multivariate logistic regression analysis identified several independent predictors of poor treatment outcomes among neonates with meningitis. Assisted vaginal delivery (AOR = 3.70; 95% CI: 1.42–9.69), positive CSF culture (AOR = 3.74; 95% CI: 1.76–7.96), elevated CSF white blood cell count >2500 cells/mm³ (AOR = 6.10; 95% CI: 2.54–14.7), high CSF protein >400 mg/dL (AOR = 5.43; 95% CI: 2.84–10.4), low CSF glucose <10 mg/dL (AOR = 3.57; 95% CI: 1.64–7.80), seizures at admission (AOR = 5.07; 95% CI: 2.71–9.46), seizures after admission (AOR = 3.88; 95% CI: 1.82–8.25), EONS (AOR = 4.27; 95% CI: 2.03–8.99), and non-exclusive breastfeeding (AOR = 2.20; 95% CI: 1.06–4.57) were significant associated with poor treatment outcomes (Table 5).

**Table 5:** Factors associated with poor outcomes among neonates with meningitis treated at public hospitals in Harar town, Eastern Ethiopia, from February 10 to March 20, 2025.

Variables	Poor outcome n (%)	Good outcome n (%)	COR (95% CI)	AOR (95% CI)	P-value
**Age (days)**					
<7	109 (40.4)	161 (59.6)	2.08 (1.42-3.05)	0.58 (0.30-1.15)	0.119
7–28	58 (24.6)	178 (75.4)	1	1	
**Birth weight (g)**					
<2500	47 (68.1)	22 (31.9)	5.64 (3.26-9.76)	2.12 (0.83-5.39)	0.115
≥2500	120 (27.5)	317 (72.5)	1	1	
**Gestational age (weeks)**					
<37	70 (53.8)	60 (46.2)	3.36 (2.22-5.08)	1.83 (0.85-3.91)	0.121
≥37	97 (25.8)	279 (74.2)	1	1	
**Mode of delivery**					
Assisted vaginal delivery (FD or VE)	20 (51.3)	19 (48.7)	2.62 (1.34-5.14)	3.70 (1.42–9.69)^a^	**0.008**
Cesarean section	57 (37.3)	96 (62.7)	1.48 (0.98-2.22)	0.80 (0.42-1.55)	0.511
Spontaneous vaginal	90 (28.7)	224 (71.3)	1	1	
**CSF culture**					
Positive	42 (59.2)	29 (40.8)	3.59 (2.14-6.02)	3.74 (1.76–7.96)^a^	**0.001**
Negative	125 (28.7)	310 (71.3)	1	1	
**CSF WBC (/mm³)**					
≥2500	55 (78.6)	15 (21.4)	10.79 (5.86-19.9)	6.10 (2.54–14.7)^b^	**<0.001**
<2500	107 (25.4)	315 (74.6)	1	1	
**CSF protein (mg/dL)**					
≥400	86 (72.9)	32 (27.1)	10.19 (6.34-16.4)	5.43 (2.84–10.4)^b^	**<0.001**
<400	81 (20.9)	307 (79.1)	1	1	
**CSF glucose (mg/dL)**					
<10	49 (62.0)	30 (38.0)	4.28 (2.59-7.06)	3.57 (1.64–7.80)^a^	**0.001**
≥10	118 (27.6)	309 (72.4)	1	1	
**Hemoglobin (g/dL)**					
<11.5	96 (48.2)	103 (51.8)	3.10 (2.11-4.55)	1.66 (0.94-2.95)	0.083
≥11.5	71 (23.1)	236 (76.9)	1	1	
15 **Platelet (×10⁹/L)**					
<150	16 (45.7)	19 (54.3)	1.79 (0.89-3.57)	0.33 (0.10-1.09)	0.069
≥150	151 (32.1)	320 (67.9)	1	1	
**Non-exclusive breastfeeding**					
Yes	35 (52.2)	32 (47.8)	2.54 (1.51-4.28)	2.20 (1.06–4.57)^a^	**0.035**
No	132 (30.1)	307 (69.9)	1	1	
**Seizure at admission**					
Yes	82 (65.6)	43 (34.4)	6.64 (4.27-10.3)	5.07 (2.71–9.46)^b^	**<0.001**
No	85 (22.3)	296 (77.7)	1	1	
**Seizure after admission**					
Yes	33 (53.2)	29 (46.8)	2.63 (1.54-4.51)	3.88 (1.82–8.25)^b^	**<0.001**
No	134 (30.2)	310 (69.8)	1	1	
**EONS**					
Yes	70 (60.3)	46 (39.7)	4.60 (2.97-7.12)	4.27 (2.03–8.99)^b^	**<0.001**
No	97 (24.9)	293 (75.1)	1	1	
**Congenital hydrocephalus**					
Yes	20 (57.1)	15 (42.9)	2.94 (1.46–5.90)	2 3.31 (0.83–6.41)	0.109
No	147 (31.2)	324 (68.8)	1	1	

Hosmer-Lemeshow goodness of fit test was fitted with a P-value of 0.53; ^a^ P < 0.05, ^b^ P < 0.001.

AOR: adjusted odds ratio; CSF: cerebrospinal fluid; C/S: cesarean section; COR: crude odds assisted vaginal delivery; ratio; EONS: early onset neonatal sepsis; FD: forceps delivery; AV: Assisted with vacuum; VE: vacuum extraction.

## Discussion

This study found that approximately one-third of neonates with meningitis experienced poor treatment outcomes, including in-hospital death or discharge with documented neurological complications, while nearly one-fifth died (18%) during hospitalization. These findings indicate that neonatal meningitis remains a major cause of morbidity and mortality in eastern Ethiopia despite the availability of neonatal intensive care services. The mortality observed in this study was lower than that reported in studies from Angola (24.0%) [23], Gambia (37.0%) [16], Tanzania (29.4%) [24], Tunisia (40.0%) [37], and India (29.1%) [38], but higher than findings from Taiwan (10.3%) [7], Colombia (9.5%) [39], and China (4.2%) [36]. The observed differences may reflect variations in healthcare infrastructure, early access to neonatal intensive care, timeliness of diagnosis and treatment, and antimicrobial resistance patterns across study settings.

Neurological sequelae were identified in 17.8% of survivors at discharge, primarily seizures and hydrocephalus. Although this proportion was comparable to reports from Tunisia (16.4%) [37], it was lower than the rates reported in studies from Canada (72.4%) [40], Taiwan (41.6%) [18], India (30.4%) [37], Nigeria (22.5%) [41], and Tunisia (21.6%) [42]. This difference should be interpreted cautiously because our assessment was limited to the period of hospitalization. Many neurodevelopmental impairments, including cognitive, hearing, language, and motor deficits, become evident only after months or years of follow-up [18,40]. Consequently, the burden of long-term disability is likely underestimated in the present study. Differences in access to neuroimaging, hearing assessment, specialist follow-up, spectrum of pathogens, and rehabilitation services may also contribute to the variability reported across studies.

Assisted vaginal delivery was independently associated with poor treatment outcomes. Because vacuum-assisted delivery is generally performed in pregnancies complicated by fetal distress, prolonged labor, or difficult delivery, the observed association is more likely to reflect underlying obstetric complications than the delivery procedure itself [36]. These complications may increase susceptibility to severe infection or neurological injury before hospital admission. In addition, exposure to healthcare-associated or antimicrobial-resistant pathogens during delivery may contribute to worse outcomes, particularly in resource-limited settings where infection prevention practices and access to timely microbiological diagnosis may be suboptimal [13,41]. Therefore, improvements in obstetric care and timely neonatal assessment may reduce subsequent adverse outcomes.

Culture-confirmed meningitis was another predictor of poor treatment outcomes. Culture positivity may indicate viable bacterial infection despite host immune responses and therefore may identify neonates with more severe disease. Previous studies conducted in China and Taiwan have reported similar associations between culture-confirmed bacterial meningitis and adverse clinical outcomes [27,43]. However, the relatively low culture positivity observed in this study may have resulted from antibiotic administration before lumbar puncture, a common challenge in routine clinical practice. Consequently, culture-negative cases should not be assumed to represent milder disease, and strengthening microbiological diagnostic capacity remains an important priority.

Severe CSF abnormalities, including markedly elevated WBC count, increased protein concentration, and profoundly reduced glucose levels, were also associated with poor treatment outcomes. These laboratory abnormalities reflect severe meningeal inflammation, blood-brain barrier disruption, and impaired glucose metabolism within the central nervous system, all of which have been linked to increased mortality and long-term neurological sequelae [27,43]. Similar observations have been reported in studies from Tunisia and Taiwan [42,43,44]. Their strong association with adverse outcomes in the present study supports the importance of comprehensive CSF analysis for early risk stratification and clinical decision-making, particularly where advanced diagnostic tools are limited.

Seizures, whether present at admission or developing during hospitalization, were also associated with poor treatment outcomes. Seizures are an important marker of severe central nervous system involvement and often indicate extensive brain injury resulting from meningitis [45]. Their occurrence after admission may also indicate progression of disease despite treatment or the development of neurological complications. This finding is consistent with studies from Canada [46], India [13], and Taiwan [43], which identified seizures as a predictor of mortality and neurological sequelae among affected neonates. This finding reinforces the need for continuous neurological monitoring, prompt seizure control, and early neuroimaging when available to minimize irreversible brain injury.

EONS was another determinant of poor treatment outcomes, supporting findings from Taiwan and Nigeria [7,41]. This association likely reflects the rapid progression of systemic infection during the first week of life and the limited physiological reserve of newborns. Early recognition and aggressive management of neonatal sepsis should therefore remain an integral component of meningitis care [27]. Similarly, non-exclusive breastfeeding was associated with poorer outcomes, reinforcing evidence that exclusive breastfeeding provides passive immune protection and reduces the severity of neonatal infections.

In contrast, prematurity, low birth weight, anemia, and thrombocytopenia were not independently associated with poor treatment outcomes after adjustment for other factors. This suggests that the severity of central nervous system infection and systemic illness may have a greater influence on prognosis than baseline neonatal characteristics. Alternatively, the effects of prematurity and low birth weight may have been mediated through other clinical variables included in the multivariable model. Because of the cross-sectional design, causal relationships cannot be established, and prospective longitudinal studies are warranted to clarify these pathways.

Overall, the findings indicate that poor outcomes in neonatal meningitis are driven primarily by severe infection and neurological involvement rather than demographic characteristics alone. Strengthening intrapartum care, ensuring prompt diagnosis of meningitis and neonatal sepsis, improving access to microbiological and CSF diagnostic services, closely monitoring high-risk neonates, and promoting exclusive breastfeeding are likely to reduce mortality and neurological complications in similar resource-limited settings.

## Conclusion

Neonatal meningitis was associated with substantial adverse outcomes. Assisted vaginal delivery, severe CSF abnormalities, seizures, EONS, and non-exclusive breastfeeding were independent predictors of poor treatment outcomes. Strengthening early detection, neonatal care, and breastfeeding promotion may improve survival and reduce neurological complications.

### Limitations and strengths of the study

This study has many main limitations. First, its retrospective cross-sectional design relied on routinely documented medical records, which limited the availability and completeness of clinical information and precluded establishing causal relationships between identified predictors and treatment outcomes. Important maternal and neonatal variables, including maternal age, education, occupation, residence, socioeconomic status, infections during pregnancy, antenatal care utilization, duration of labour, vaginal discharge, spina bifida, and prior antibiotic exposure, and clinical factors such as the sensitivity pattern of the isolate pathogens and blood glucose, were incompletely documented and therefore could not be assessed.

Secondly, the evaluation of treatment outcomes only considered the period of hospitalization. As a result, the study could not assess long-term neurodevelopmental outcomes. These outcomes include cognitive impairments, behavioral problems, or delays in motor and language development, which may only show up months or years later. Third, the study did not differentiate between community-acquired and hospital-acquired infections. This distinction is important because the organisms causing these infections, their resistance patterns, and their prognostic implications often differ. Fourth, excluding neonates with incomplete medical records may have introduced selection bias. Consequently, the true frequency of poor treatment outcomes may have been underestimated.

Fifth, the study was conducted in only two public referral hospitals in eastern Ethiopia. Although these hospitals provide care for a large number of neonates, the findings may not be generalizable to other healthcare settings, including primary or private hospitals.

Finally, the timing of lumbar puncture relative to the initiation of empirical antibiotic therapy was not consistently documented in the medical records. This may have reduced cerebrospinal fluid culture yield and led to an underestimation of microbiologically confirmed meningitis cases.

Despite these limitations, the study has important strengths. It included a relatively modest sample of neonates from two major public hospitals. These strengths improve the reliability of the findings and provide valuable evidence on treatment outcomes and their associated factors in a resource-limited setting where such data are scarce.
