# Supplementary material for: Treatment outcomes of meningitis and its associated factors among neonates admitted to public hospitals in Harar Town, Ethiopia: Cross-sectional study
Source: PLoS One. 2026 Aug 11;21(8):e0355843. doi: 10.1371/journal.pone.0355843 (PMC13460571; doi:10.1371/journal.pone.0355843)
Supplement: Supplementary file 1 [file pone.0355843.s002.docx]

**Response to the Academic Editor**

Dear **Dr. Dominic Umoru**,

We sincerely thank you for your careful evaluation of our manuscript and for providing us with the opportunity to revise and resubmit it to **PLOS ONE**. We are grateful for your time and for the constructive comments and suggestions provided by you and the reviewers. These insightful comments have been invaluable in improving the quality, clarity, and scientific rigor of our manuscript.

We have carefully considered and addressed each comment and revised the manuscript accordingly. A detailed, point-by-point response to all comments is provided in the accompanying response document, and all revisions have been highlighted in the revised manuscript for ease of review.

The major revisions include clarification of the study methodology and revision of the discussion, limitations, and conclusions. In addition, the reference list was thoroughly reviewed and revised to ensure accuracy and consistency. We updated the references to reflect the revised manuscript, corrected formatting where necessary, ensured consistency between the in-text citations and the reference list, and verified that none of the cited articles have been retracted.

We believe that these revisions have substantially strengthened the manuscript and have satisfactorily addressed all of the concerns raised during the review process.

Thank you for your time, thoughtful consideration, and the opportunity to improve our work. We appreciate your continued consideration of our revised manuscript and look forward to your decision.

Sincerely,

**Getahun Tamiru Yirsaw**

Corresponding Author

Clinical Pharmacy Unit, Haramaya University

Harar, Ethiopia

Dear **Reviewers**

We sincerely thank the reviewers for their careful evaluation of our manuscript and for their positive assessment. We greatly appreciate that both reviewers found the study to be technically sound, the statistical analyses appropriate, the conclusions supported by the data, the underlying data adequately available, and the manuscript written in clear and intelligible English.

We are also grateful for the constructive comments and suggestions provided, which have helped us further improve the quality and clarity of the manuscript. We have carefully addressed each comment in a point-by-point manner below, and all corresponding revisions have been incorporated into the revised manuscript and highlighted for ease of review.

Sincerely,

**Getahun Tamiru Yirsaw**

Corresponding Author

Clinical Pharmacy Unit, Haramaya University

Harar, Ethiopia

**Reviewer #1**

**Comment 1:** Was any of the deliveries carried out by forceps delivery? I see no mention of that as one of the modes of delivery.

**Response:** Thank you for this valuable comment. Forceps-assisted delivery was inadvertently grouped under assisted vaginal delivery in the original manuscript. Because the number of forceps- and vacuum-assisted deliveries was small, they were combined into a single category (**assisted vaginal delivery**) to ensure adequate statistical power for the analysis. Based on your suggestion, we have clarified this classification in the Methods section and revised the Results, Tables, and Discussion accordingly.

**Comment 2:** Outcome measurement; please make this explicit about timing of the measurement (at time of discharge or death), as this shows short-term/immediate outcomes.

**Response:** Thank you for this important comment. We agree that the timing of outcome assessment was not explicitly stated in the original manuscript. We have now clarified that treatment outcomes were assessed at **the time of hospital discharge or in-hospital death,** reflecting short-term (in-hospital) outcomes. This clarification has been added to the Outcome Measurement section of the Methods.

**Comment 3:** What is your definition of ‘confirmed meningitis’? Did all neonates have lumbar puncture with positive cultures or biochemistry? Were antibiotics started before lumbar puncture or after?

**Response:** Thank you for this important comment. We have revised the Methods section to define neonatal meningitis according to diagnostic certainty (confirmed, probable, and suspected). We also clarified that all included neonates underwent lumbar puncture as part of the diagnostic evaluation and not all cases were confirmed. **Confirmed meningitis** was defined by identification of a causative pathogen in the cerebrospinal fluid by culture and/or Gram stain. The exact timing of lumbar puncture relative to initiation of empirical antibiotic therapy was not consistently documented in the medical records and therefore could not be evaluated. This limitation has been acknowledged in the revised manuscript.

**Comment 4**

**Results: Recast the heading to "Clinical findings, treatment and outcome".**

**Response:** Thank you for this helpful suggestion. We have revised the Results section heading to **"Clinical findings, treatment and outcomes"** and relocated the relevant clinical variables to this section to improve the organization of the manuscript.

**Comment 5**

**Assisted vaginal delivery was linked to poor outcomes... explain why prematurity and low birth weight did not show an effect.**

**Response:** Thank you for this insightful comment. We revised the Discussion to clarify that although assisted vaginal delivery was independently associated with poor treatment outcomes, the underlying mechanism cannot be established in this retrospective observational study. We therefore interpret this association cautiously and acknowledge that it may reflect underlying obstetric complications rather than a direct causal effect. We also expanded the Discussion to explain that prematurity and low birth weight did not remain significant in the multivariable model, possibly because their effects were mediated through other clinical factors or because of confounding.

**Comment 6**

**Table 4: "Discharge without improvement" is unclear.**

**Response:** Thank you for pointing this out. "Discharge without improvement" referred to neonates whose caregivers requested discharge against medical advice before clinical improvement. We have clarified this definition in the table footnote and the Results section.

**Comment 7**

**State that the study is retrospective.**

**Response:** Thank you. We have explicitly stated in the Limitations that this was a retrospective cross-sectional study based on medical record review.

**Comment 8**

**Selection bias due to exclusion of incomplete records.**

**Response:** We appreciate this important observation. We have expanded the Limitations to acknowledge that excluding neonates with incomplete medical records may have introduced selection bias because severely ill neonates often have incomplete documentation. This may have resulted in underestimation of poor treatment outcomes.

**Comment 9**

**Study limited to two hospitals.**

**Response:** Thank you for this comment. We have added this limitation, noting that the study was conducted in two public hospitals in Harar Town, which may limit the generalizability of the findings to other healthcare settings in Ethiopia.

**Comment 10**

Was there data on platelet (and other variables such as Hb count) for all the subjects? If not, what was the denominator for concluding that 6.9% had thrombocytopaenia, for example?

**Response:**

Thank you for this important question. We confirm that laboratory investigations, including platelet count and hemoglobin levels, were available for all neonates included in the study. Therefore, the denominator used for calculating thrombocytopenia and other hematological abnormalities was the total study population.

**Comment 11**

Please, can you confirm that all the neonates included in the study had a hearing test done on them? Clarify how the hearing loss 0.2% was arrived at.

**Response:**
Thank you for this valuable comment. We confirm that hearing assessment was not performed for all neonates included in the study. Only one neonate had a documented hearing test result indicating hearing loss. Therefore, the reported prevalence of hearing loss (0.2%) was calculated based on the total number of study participants, with 1 case of hearing loss among the full study.

**Response to Reviewer #2**

**Comment 1:** Table 1 is deficient with place of delivery as it pertains to the socio-demography of the baby.

**Response:** Thank you for this valuable suggestion. We agree that place of delivery is an important characteristic. We have revised Table 1 to include the place of delivery, as this information was available in the medical records.

**Comment 2:** The socio-demographic characteristics of the parents, especially the mother, are also important and need to be included.

**Response:** We agree that parental, particularly maternal, socio-demographic characteristics are important determinants of neonatal health. However, because this was a retrospective study based on neonatal medical records, maternal socio-demographic information (such as maternal age, education, occupation, and residence) was not routinely documented and was therefore unavailable for analysis. We have acknowledged this limitation in the revised manuscript.

**Comment 3:** Table 2 should also have highlighted if there was any spina bifida, duration of labour, and presence or absence of vaginal discharge.

**Response:** Thank you for this important suggestion. We agree that spina bifida, duration of labour, and maternal vaginal discharge are clinically relevant factors. We reviewed all eligible medical records and found that no neonates had a documented diagnosis of spina bifida. Therefore, they were not included in Table 2. We have acknowledged the lack of data on spina bifida as a limitation of the study

**Comment 4:** Sensitivity pattern of the isolate is not indicated. CSF glucose is interpreted in relation to blood glucose.

**Response:** Thank you for this important comment. We agree that antimicrobial susceptibility patterns and the CSF-to-blood glucose ratio provide valuable clinical information. However, because this was a retrospective chart review, antimicrobial susceptibility testing results and simultaneous blood glucose measurements were not consistently documented in the medical records and therefore could not be analyzed reliably. This limitation has been acknowledged in the revised manuscript.

**Comment 5**

**What is the clear difference between status of the patient at discharge and treatment outcomes? I suggest merging.**

**Response:** We appreciate this helpful suggestion and agree that the two variables overlapped conceptually. Accordingly, we merged **patient status at discharge** with **treatment outcomes** to improve clarity and avoid redundancy. The corresponding table and Results section have been revised accordingly.

**Comment 6:** Even though this is a retrospective study, no data were presented on the relationship between antibiotic combinations and treatment outcomes.

**Response:** Thank you for this valuable suggestion. We agree that examining the association between empirical antibiotic regimens and treatment outcomes would strengthen the study. However, treatment regimens were determined by hospital protocol and only a small proportion received alternative regimens, the study may have been underpowered to detect differences. We conducted an additional analysis to explore the relationship between antibiotic regimens and treatment outcomes. No statistically significant association was observed between the different empirical antibiotic combinations and treatment outcomes.

**Comment 7**

**The first paragraph in the discussion is confusing. Is the 18.8% observed mortality rate higher than 24% in Angola and 37% in Gambia? Clarify.**

**Response:** We appreciate the reviewer's careful reading. We revised the first paragraph of the Discussion to accurately compare our findings with previous studies. The wording has been corrected to clearly indicate that the observed mortality rate of 18.8% was **lower** than the reported mortality rates of 24% in Angola and 37% in The Gambia.

**Comment 8**

**The discussion lacks critical appraisal of the observations and discordance with other studies and the reasons for such observations and discordance.**

**Response:** Thank you for this valuable recommendation. We substantially revised the Discussion by providing a more critical interpretation of our findings, comparing them with previous studies, and discussing possible reasons for similarities and differences, including variations in study populations, healthcare settings, diagnostic practices, disease severity, and treatment protocols.

**Comment 9**

**Conclusion is not focused, even as the first sentence is a repetition of the opening statement of the discussion.**

**Response:** We appreciate this suggestion. The Conclusion has been rewritten to improve focus and conciseness. Repetitive statements have been removed, and the section now emphasizes the principal findings, their clinical implications, and recommendations for future research.
