# Supplementary material for: Treatment outcomes of meningitis and its associated factors among neonates admitted to public hospitals in Harar Town, Ethiopia: Cross-sectional study
Source: PLoS One. 2026 Aug 11;21(8):e0355843. doi: 10.1371/journal.pone.0355843 (PMC13460571; doi:10.1371/journal.pone.0355843)
Supplement: Peer review history

# Peer review: Decision Letter 0

**DOI:** 10.1371/journal.pone.0355843.r001

**Authors:** Dominic Umoru

28 Jun 2026

Dear Dr. Yirsaw,

Line 231: Was there data on platelet (and other variables such as Hb count for all the subjects? If not, what was the denominator for concluding that 6.9% had thrombocytopaenia, for example?

Line 240-244: Please, can you confirm that all the neonates included in the study had a hearing test done on them? Clarify how the hearing loss 0.2% was arrived at.

Line 268-374: First paragraph under Discussion needs complete revision. For example, 18.8% (study location) cannot be higher than 24% (Angola). For conformity, keep all figures at one decimal point (e.g., 24.0) throughout the document.

Line 333: You may wish to correct "Second" to "Secondly"

Some abbreviations were used and then defined later in the document. Define EONS, LONS, and other abbreviations when first used.

Please submit your revised manuscript by Aug 12 2026 11:59PM. If you will need more time than this to complete your revisions, please reply to this message or contact the journal office at plosone@plos.org. When you're ready to submit your revision, log on to https://www.editorialmanager.com/pone/ and select the 'Submissions Needing Revision' folder to locate your manuscript file.

- A letter that responds to each point raised by the academic editor and reviewer(s). You should upload this letter as a separate file labeled 'Response to Reviewers'.
- A marked-up copy of your manuscript that highlights changes made to the original version. You should upload this as a separate file labeled 'Revised Manuscript with Track Changes'.
- An unmarked version of your revised paper without tracked changes. You should upload this as a separate file labeled 'Manuscript'.

If you would like to make changes to your financial disclosure, please include your updated statement in your cover letter. Guidelines for resubmitting your figure files are available below the reviewer comments at the end of this letter.

If applicable, we recommend that you deposit your laboratory protocols in protocols.io to enhance the reproducibility of your results. Protocols.io assigns your protocol its own identifier (DOI) so that it can be cited independently in the future. For instructions see: https://journals.plos.org/plosone/s/submission-guidelines#loc-laboratory-protocols. Additionally, PLOS ONE offers an option for publishing peer-reviewed Lab Protocol articles, which describe protocols hosted on protocols.io. Read more information on sharing protocols at https://plos.org/protocols?utm_medium=editorial-email&utm_source=authorletters&utm_campaign=protocols.

As the corresponding author, your ORCID iD is verified in the submission system and will appear in the published article. PLOS supports the use of ORCID, and we encourage all coauthors to register for an ORCID iD and use it as well. Please encourage your coauthors to verify their ORCID iD within the submission system before final acceptance, as unverified ORCID iDs will not appear in the published article. *Only*  the individual author can complete the verification step; PLOS staff *cannot*  verify ORCID iDs on behalf of authors.

We look forward to receiving your revised manuscript.

Kind regards,

Dominic Umoru, MBBS, MSc, FWACP, MRCPCH

Academic Editor

PLOS One

Journal Requirements:

When submitting your revision, we need you to address these additional requirements.

1. Please ensure that your manuscript meets PLOS ONE's style requirements, including those for file naming. The PLOS ONE style templates can be found at

https://journals.plos.org/plosone/s/file?id=wjVg/PLOSOne_formatting_sample_main_body.pdf and

https://journals.plos.org/plosone/s/file?id=ba62/PLOSOne_formatting_sample_title_authors_affiliations.pdf

2. Please include a caption for figure 1.

3. If the reviewer comments include a recommendation to cite specific previously published works, please review and evaluate these publications to determine whether they are relevant and should be cited. There is no requirement to cite these works unless the editor has indicated otherwise.

4. Please review your reference list to ensure that it is complete and correct. If you have cited papers that have been retracted, please include the rationale for doing so in the manuscript text, or remove these references and replace them with relevant current references. Any changes to the reference list should be mentioned in the rebuttal letter that accompanies your revised manuscript. If you need to cite a retracted article, indicate the article’s retracted status in the References list and also include a citation and full reference for the retraction notice.

[Note: HTML markup is below. Please do not edit.]

Reviewers' comments:

Reviewer's Responses to Questions

**Comments to the Author**

1. Is the manuscript technically sound, and do the data support the conclusions?

Reviewer #1: Yes

Reviewer #2: Yes

**********

2. Has the statistical analysis been performed appropriately and rigorously?

Reviewer #1: Yes

Reviewer #2: Yes

**********

3. Have the authors made all data underlying the findings in their manuscript fully available?

The PLOS Data policy

Reviewer #1: Yes

Reviewer #2: Yes

**********

4. Is the manuscript presented in an intelligible fashion and written in standard English?

Reviewer #1: Yes

Reviewer #2: Yes

**********

Reviewer #1: Authors have presented a cross-sectional study from assessing treatment outcomes of neonatal meningitis and identifying factors associated with poor prognosis among 506 neonates admitted to two public hospitals in Harar, Ethiopia, between 2020 and 2024. The study aimed to determine which clinical and laboratory factors predicted these outcomes.

Introduction

Method: line 121 - was any of the deliveries carried out by forceps delivery? I see no mention of that as one of the modes of delivery.

Outcome measurement; please make this explicit with regards to timing of the measurement ’’outcome measurement was as at time of discharge or death’’. This will show you were looking at short term/immediate outcomes as some of the outcomes could be long term

What is your definition of ‘confirmed meningitis’? Did all neonates have lumbar puncture with positive cultures or biochemistry? Were antibiotics started before lumbar puncture or after-need to state that.

Results: Line 239 - I suggest you recast the heading to ‘’Clinical findings, treatment and outcome’’ to replace ‘’prescribed medications and outcome of treatment’’. Then include lines 237 & 238 in this section instead of under ‘’

laboratory and clinical findings of patients’’

Assisted vaginal delivery was linked to poor outcomes, but the reason for this is not clear, so you should either provide stronger evidence or say that the explanation is uncertain. You should also explain why prematurity and low birth weight did not show an effect in your analysis, even though they are usually known risk factors for poor outcome

Table 4 under ‘’status of patients at discharge’’ under catergory Discharge without improvement – this is not clear as one is left wondering why discharge a patient if they were not improving. Did parents self discharge?

Discussion:

Limitation: Study is retrospective, this should be stated.

As you excluded neonates with missing data, there is a risk of selection bias since the sickest babies often have incomplete charts. This could make the study underestimate how many poor outcomes truly occurred, and this limitation should be clearly acknowledged.

You should add the study being limited to 2 hospital makes generalization difficult. Excluding neonates with missing data, is a risk of selection bias since the sickest babies often have incomplete charts. This could make the study underestimate how many poor outcomes truly occurred, and this limitation should be clearly acknowledged.

Reviewer #2: This study is a retrospective study with some pertinent information which is beyond the authors capacity to provide if not documented. However, Table 1 is deficient with place of delivery as it pertains to the socio-demography of the baby.

The socio-demographic characteristics of the parents especially the mother is also important and needs to be included.

Table 2 should also have highlighted if there was any spinal bifida, duration of labour and presence or absence of vaginal discharge

Sensitivity pattern of the isolate is not indicated

CSF glucose is interpreted in relation to blood glucose. Even though this is a retrospective study no single data on this was highlighted

Relationship between the good and bad outcomes was not reviewed in relation to the combinations of antibiotics administered

Whats the clear difference between between status of the patient at discharge and treatment outcomes. Is the status at the end of treatment not same as treatment outcomes. I suggest merging.

Why is spina bifida not included as type of pre-existing medical condition.

The first paragraph in the discussion is confusing. Is the 18.8% observed mortality rate higher than 24% in Angola, 37% in Gambia. Clarify?

The discussions lack critical appraisal of the observations and discordance with other studies and the reasons for such observations and discordance.

Conclusion is not focused, even as the first sentence is a repetition of the opening statement of the discussion.

**********

what does this mean?). If published, this will include your full peer review and any attached files.

If you choose “no”, your identity will remain anonymous but your review may still be made public.

**Do you want your identity to be public for this peer review?**  For information about this choice, including consent withdrawal, please see our Privacy Policy

Reviewer #1: **Yes:**  Francis Akor

Reviewer #2: **Yes:**  Ochigbo Sunday

**********

[NOTE: If reviewer comments were submitted as an attachment file, they will be attached to this email and accessible via the submission site. Please log into your account, locate the manuscript record, and check for the action link "View Attachments". If this link does not appear, there are no attachment files.]

To ensure your figures meet our technical requirements, please review our figure guidelines: https://journals.plos.org/plosone/s/figures

You may also use PLOS’s free figure tool, NAAS, to help you prepare publication quality figures: https://journals.plos.org/plosone/s/figures#loc-tools-for-figure-preparation.

NAAS will assess whether your figures meet our technical requirements by comparing each figure against our figure specifications.

# Author comment: Author response to Decision Letter 1

**DOI:** 10.1371/journal.pone.0355843.r002

24 Jul 2026

Response to the Academic Editor

Dear Dr. Dominic Umoru,

We sincerely thank you for your careful evaluation of our manuscript and for providing us with the opportunity to revise and resubmit it to PLOS ONE. We are grateful for your time and for the constructive comments and suggestions provided by you and the reviewers. These insightful comments have been invaluable in improving the quality, clarity, and scientific rigor of our manuscript.

We have carefully considered and addressed each comment and revised the manuscript accordingly. A detailed, point-by-point response to all comments is provided in the accompanying response document, and all revisions have been highlighted in the revised manuscript for ease of review.

The major revisions include clarification of the study methodology and revision of the discussion, limitations, and conclusions. In addition, the reference list was thoroughly reviewed and revised to ensure accuracy and consistency. We updated the references to reflect the revised manuscript, corrected formatting where necessary, ensured consistency between the in-text citations and the reference list, and verified that none of the cited articles have been retracted.

We believe that these revisions have substantially strengthened the manuscript and have satisfactorily addressed all of the concerns raised during the review process.

Thank you for your time, thoughtful consideration, and the opportunity to improve our work. We appreciate your continued consideration of our revised manuscript and look forward to your decision.

Sincerely,

Getahun Tamiru Yirsaw

Corresponding Author

Clinical Pharmacy Unit, Haramaya University

Harar, Ethiopia

Dear Reviewers

We sincerely thank the reviewers for their careful evaluation of our manuscript and for their positive assessment. We greatly appreciate that both reviewers found the study to be technically sound, the statistical analyses appropriate, the conclusions supported by the data, the underlying data adequately available, and the manuscript written in clear and intelligible English.

We are also grateful for the constructive comments and suggestions provided, which have helped us further improve the quality and clarity of the manuscript. We have carefully addressed each comment in a point-by-point manner below, and all corresponding revisions have been incorporated into the revised manuscript and highlighted for ease of review.

Sincerely,

Getahun Tamiru Yirsaw

Corresponding Author

Clinical Pharmacy Unit, Haramaya University

Harar, Ethiopia

Reviewer #1

Comment 1: Was any of the deliveries carried out by forceps delivery? I see no mention of that as one of the modes of delivery.

Response: Thank you for this valuable comment. Forceps-assisted delivery was inadvertently grouped under assisted vaginal delivery in the original manuscript. Because the number of forceps- and vacuum-assisted deliveries was small, they were combined into a single category (assisted vaginal delivery) to ensure adequate statistical power for the analysis. Based on your suggestion, we have clarified this classification in the Methods section and revised the Results, Tables, and Discussion accordingly.

Comment 2: Outcome measurement; please make this explicit about timing of the measurement (at time of discharge or death), as this shows short-term/immediate outcomes.

Response: Thank you for this important comment. We agree that the timing of outcome assessment was not explicitly stated in the original manuscript. We have now clarified that treatment outcomes were assessed at the time of hospital discharge or in-hospital death, reflecting short-term (in-hospital) outcomes. This clarification has been added to the Outcome Measurement section of the Methods.

Comment 3: What is your definition of ‘confirmed meningitis’? Did all neonates have lumbar puncture with positive cultures or biochemistry? Were antibiotics started before lumbar puncture or after?

Response: Thank you for this important comment. We have revised the Methods section to define neonatal meningitis according to diagnostic certainty (confirmed, probable, and suspected). We also clarified that all included neonates underwent lumbar puncture as part of the diagnostic evaluation and not all cases were confirmed. Confirmed meningitis was defined by identification of a causative pathogen in the cerebrospinal fluid by culture and/or Gram stain. The exact timing of lumbar puncture relative to initiation of empirical antibiotic therapy was not consistently documented in the medical records and therefore could not be evaluated. This limitation has been acknowledged in the revised manuscript.

Comment 4

Results: Recast the heading to "Clinical findings, treatment and outcome".

Response: Thank you for this helpful suggestion. We have revised the Results section heading to "Clinical findings, treatment and outcomes" and relocated the relevant clinical variables to this section to improve the organization of the manuscript.

Comment 5

Assisted vaginal delivery was linked to poor outcomes... explain why prematurity and low birth weight did not show an effect.

Response: Thank you for this insightful comment. We revised the Discussion to clarify that although assisted vaginal delivery was independently associated with poor treatment outcomes, the underlying mechanism cannot be established in this retrospective observational study. We therefore interpret this association cautiously and acknowledge that it may reflect underlying obstetric complications rather than a direct causal effect. We also expanded the Discussion to explain that prematurity and low birth weight did not remain significant in the multivariable model, possibly because their effects were mediated through other clinical factors or because of confounding.

Comment 6

Table 4: "Discharge without improvement" is unclear.

Response: Thank you for pointing this out. "Discharge without improvement" referred to neonates whose caregivers requested discharge against medical advice before clinical improvement. We have clarified this definition in the table footnote and the Results section.

Comment 7

State that the study is retrospective.

Response: Thank you. We have explicitly stated in the Limitations that this was a retrospective cross-sectional study based on medical record review.

Comment 8

Selection bias due to exclusion of incomplete records.

Response: We appreciate this important observation. We have expanded the Limitations to acknowledge that excluding neonates with incomplete medical records may have introduced selection bias because severely ill neonates often have incomplete documentation. This may have resulted in underestimation of poor treatment outcomes.

Comment 9

Study limited to two hospitals.

Response: Thank you for this comment. We have added this limitation, noting that the study was conducted in two public hospitals in Harar Town, which may limit the generalizability of the findings to other healthcare settings in Ethiopia.

Comment 10

Was there data on platelet (and other variables such as Hb count) for all the subjects? If not, what was the denominator for concluding that 6.9% had thrombocytopaenia, for example?

Response:

Thank you for this important question. We confirm that laboratory investigations, including platelet count and hemoglobin levels, were available for all neonates included in the study. Therefore, the denominator used for calculating thrombocytopenia and other hematological abnormalities was the total study population.

Comment 11

Please, can you confirm that all the neonates included in the study had a hearing test done on them? Clarify how the hearing loss 0.2% was arrived at.

Response:

Thank you for this valuable comment. We confirm that hearing assessment was not performed for all neonates included in the study. Only one neonate had a documented hearing test result indicating hearing loss. Therefore, the reported prevalence of hearing loss (0.2%) was calculated based on the total number of study participants, with 1 case of hearing loss among the full study.

Response to Reviewer #2

Comment 1: Table 1 is deficient with place of delivery as it pertains to the socio-demography of the baby.

Response: Thank you for this valuable suggestion. We agree that place of delivery is an important characteristic. We have revised Table 1 to include the place of delivery, as this information was available in the medical records.

Comment 2: The socio-demographic characteristics of the parents, especially the mother, are also important and need to be included.

Response: We agree that parental, particularly maternal, socio-demographic characteristics are important determinants of neonatal health. However, because this was a retrospective study based on neonatal medical records, maternal socio-demographic information (such as maternal age, education, occupation, and residence) was not routinely documented and was therefore unavailable for analysis. We have acknowledged this limitation in the revised manuscript.

Comment 3: Table 2 should also have highlighted if there was any spina bifida, duration of labour, and presence or absence of vaginal discharge.

Response: Thank you for this important suggestion. We agree that spina bifida, duration of labour, and maternal vaginal discharge are clinically relevant factors. We reviewed all eligible medical records and found that no neonates had a documented diagnosis of spina bifida. Therefore, they were not included in Table 2. We have acknowledged the lack of data on spina bifida as a limitation of the study

Comment 4: Sensitivity pattern of the isolate is not indicated. CSF glucose is interpreted in relation to blood glucose.

Response: Thank you for this important comment. We agree that antimicrobial susceptibility patterns and the CSF-to-blood glucose ratio provide valuable clinical information. However, because this was a retrospective chart review, antimicrobial susceptibility testing results and simultaneous blood glucose measurements were not consistently documented in the medical records and therefore could not be analyzed reliably. This limitation has been acknowledged in the revised manuscript.

Comment 5

What is the clear difference between status of the patient at discharge and treatment outcomes? I suggest merging.

Response: We appreciate this helpful suggestion and agree that the two variables overlapped conceptually. Accordingly, we merged patient status at discharge with treatment outcomes to improve clarity and avoid redundancy. The corresponding table and Results section have been revised accordingly.

Comment 6: Even though this is a retrospective study, no data were presented on the relationship between antibiotic combinations and treatment outcomes.

Response: Thank you for this valuable suggestion. We agree that examining the association between empirical antibiotic regimens and treatment outcomes would strengthen the study. However, treatment regimens were determined by hospital protocol and only a small proportion received alternative regimens, the study may have been underpowered to detect differences. We conducted an additional analysis to explore the relationship between antibiotic regimens and treatment outcomes. No statistically significant association was observed between the different empirical antibiotic combinations and treatment outcomes.

Comment 7

The first paragraph in the discussion is confusing. Is the 18.8% observed mortality rate higher than 24% in Angola and 37% in Gambia? Clarify.

Response: We appreciate the reviewer's careful reading. We revised the first paragraph of the Discussion to accurately compare our findings with previous studies. The wording has been corrected to clearly indicate that the observed mortality rate of 18.8% was lower than the reported mortality rates of 24% in Angola and 37% in The Gambia.

Comment 8

The discussion lacks critical appraisal of the observations and discordance with other studies and the reasons for such observations and discordance.

Response: Thank you for this valuable recommendation. We substantially revised the Discussion by providing a more critical interpretation of our findings, comparing them with previous studies, and discussing possible reasons for similarities and differences, including variations in study populations, healthcare settings, diagnostic practices, disease severity, and treatment protocols.

Comment 9

Conclusion is not focused, even as the first sentence is a repetition of the opening statement of the discussion.

Response: We appreciate this suggestion. The Conclusion has been rewritten to improve focus and conciseness. Repetitive statements have been removed, and the section now emphasizes the principal findings, their clinical implications, and recommendations for future research.

Attachment Submitted filename: Response to Reviewers.docx

# Editor report: Decision Letter 1

**DOI:** 10.1371/journal.pone.0355843.r003

**Authors:** Dominic Umoru

28 Jul 2026

Treatment Outcomes of Meningitis and Its Associated Factors among Neonates Admitted to Public Hospitals in Harar Town, Ethiopia: Cross-sectional study

PONE-D-26-10805R1

Dear Getahun Tamiru,

We’re pleased to inform you that your manuscript has been judged scientifically suitable for publication and will be formally accepted for publication once it meets all outstanding technical requirements.

Within one week, you’ll receive an e-mail detailing the required amendments. When these have been addressed, you’ll receive a formal acceptance letter, and your manuscript will be scheduled for publication.

An invoice will be generated when your article is formally accepted. Please note that if your institution has a publishing partnership with PLOS and your article meets the relevant criteria, all or part of your publication costs will be covered. Please make sure your user information is up-to-date by logging into Editorial Manager at Editorial Manager® and clicking the ‘Update My Information' link at the top of the page. For questions related to billing, please contact billing support.

If your institution or institutions have a press office, please notify them about your upcoming paper to help maximize its impact. If they’ll be preparing press materials, please inform our press team as soon as possible -- no later than 48 hours after receiving the formal acceptance. Your manuscript will remain under strict press embargo until 2 pm Eastern Time on the date of publication. For more information, please contact onepress@plos.org.

Kind regards,

Dominic Umoru, MBBS, MSc (Pub. Hlth), FWACP (Paed), MRCPCH (UK)

Academic Editor

PLOS One

Additional Editor Comments (optional):

Reviewers' comments:

# Editor report: Acceptance letter

**DOI:** 10.1371/journal.pone.0355843.r004

**Authors:** Dominic Umoru

PONE-D-26-10805R1

PLOS One

Dear Dr. Yirsaw,

I'm pleased to inform you that your manuscript has been deemed suitable for publication in PLOS One. Congratulations! Your manuscript is now being handed over to our production team.

At this stage, our production department will prepare your paper for publication. This includes ensuring the following:

* All references, tables, and figures are properly cited

* All relevant supporting information is included in the manuscript submission,

* There are no issues that prevent the paper from being properly typeset

You will receive further instructions from the production team, including instructions on how to review your proof when it is ready. Please keep in mind that we are working through a large volume of accepted articles, so please give us a few days to review your paper and let you know the next and final steps.

Lastly, if your institution or institutions have a press office, please let them know about your upcoming paper now to help maximize its impact. If they'll be preparing press materials, please inform our press team within the next 48 hours. Your manuscript will remain under strict press embargo until 2 pm Eastern Time on the date of publication. For more information, please contact onepress@plos.org.

You will receive an invoice from PLOS for your publication fee after your manuscript has reached the completed accept phase. If you receive an email requesting payment before acceptance or for any other service, this may be a phishing scheme. Learn how to identify phishing emails and protect your accounts at https://explore.plos.org/phishing.

If we can help with anything else, please email us at customercare@plos.org.

Thank you for submitting your work to PLOS ONE and supporting open access.

Kind regards,

PLOS ONE Editorial Office Staff

on behalf of

Dr. Dominic Umoru

Academic Editor

PLOS One
